# Predicting vasovagal reactions to needles with anticipatory facial temperature profiles

**DOI:** 10.1038/s41598-023-36207-z

**Published:** 2023-06-14

**Authors:** Judita Rudokaite, L. L. Sharon Ong, Itir Onal Ertugrul, Mart P. Janssen, Elisabeth M. J. Huis in ‘t Veld

**Affiliations:** 1 Department of Cognitive Science and Artificial Intelligence, Tilburg University, Tilburg, The Netherlands; 2 Donor Medicine Research, Sanquin Research, Amsterdam, The Netherlands; 3 Department of Information and Computing Sciences, Utrecht University, Utrecht, The Netherlands; 4 Department of Cognitive Science & Artificial Intelligence, Tilburg University, PO Box 90153, Warandelaan 2 (Room D147), 5000 LE Tilburg, The Netherlands

**Keywords:** Neurophysiology, Human behaviour

## Abstract

Around one-third of adults are scared of needles, which can result in adverse emotional and physical responses such as dizziness and fainting (e.g. vasovagal reactions; VVR) and consequently, avoidance of healthcare, treatments, and immunizations. Unfortunately, most people are not aware of vasovagal reactions until they escalate, at which time it is too late to intervene. This study aims to investigate whether facial temperature profiles measured in the waiting room, prior to a blood donation, can be used to classify who will and will not experience VVR during the donation. Average temperature profiles from six facial regions were extracted from pre-donation recordings of 193 blood donors, and machine learning was used to classify whether a donor would experience low or high levels of VVR during the donation. An XGBoost classifier was able to classify vasovagal groups from an adverse reaction during a blood donation based on this early facial temperature data, with a sensitivity of 0.87, specificity of 0.84, F1 score of 0.86, and PR-AUC of 0.93. Temperature fluctuations in the area under the nose, chin and forehead have the highest predictive value. This study is the first to demonstrate that it is possible to classify vasovagal responses during a blood donation using temperature profiles.

## Introduction

In 2020, the world sighed with relief when effective vaccines for the SARS-cov-2 virus were brought to market. Unfortunately, the opposite was true for the 20–50% of adults and 60% of adolescents and children suffering from needle fear^1,2^. Needle fear and associated physical reactions are highly adverse and can cause avoidance of healthcare and immunizations^2–6^, resulting in worse public and individual outcomes.

Needle fear is not easily solved^7^, as merely the sight of needles can cause extreme emotional (e.g., fear, panic) and physical reactions such as nausea, dizziness, fainting (vasovagal reactions; VVR)^8–10^. In anticipation to a needle-related event such as a blood donation, it is possible to measure rising levels of psychological, physiological and hormonal stress levels, which then peak when the needle is (about to be) inserted^11–13^. Why exactly these reactions occur is unclear. Some VVR such as sweating, changes in facial pallor, nausea, pupillary dilation, heart palpitations, and hyperventilation, are associated with unconscious autonomic activations. In contrast, others seem to occur due to increased parasympathetic activity, such as drops in heart rate and blood pressure, and increased vasodilation. Additionally, symptoms of hypoperfusion, such as light-headedness and blurred vision are observed^14,15^. Possibly, these can be explained by the activation of extended neural networks that occur during the perception of needles, which are related to the anticipation and the experience of pain, and the regulation of actions, emotions and their resulting physiological states^16,17^. Consequently, these processes could result in activation of both the sympathetic and parasympathetic nervous systems^18^.

Research on needle fear and vasovagal reactions have mostly been done in the context of blood donations, which highlighted the existence of quite a few risk-factors, such as being female, younger age, donation experience^19^, previous occurrence of a VVR^20^, waiting time^21^, ambient temperature and humidity^22^. Though informative, these do not allow a real-time, continuous way to monitor the ongoing physical and psychological states of an individual about to undergo a needle-related procedure. Another challenge in the early signaling and prevention of VVR is that people are often unable to self-report them, or they can only do so only when it is too late to prevent these from happening^23^. Even though there are multiple markers of autonomous nervous system activity that could serve as potential VVR markers, such as blood pressure, heart rate or hormones^12,22,24–27^, we specifically aim to assess whether it is possible to find a measure which could provide an immediate result on-site in a clinical setting, but which does not rely on external devices, measurements on a sample of saliva or blood afterwards, or trained personnel. Thus, in this study we propose to use a novel and non-invasive video based technique called Infrared Thermal Imaging (ITI). ITI can be used to measure minute local changes in human body temperature, which may be influenced by many internal and external factors such as metabolic activity, vasoconstriction, and sympathetic and parasympathetic activities^28^. Specifically, previous studies showed that ITI measurement is a successful technique for predicting people’s psychological stress^29,30^ or overall emotional and psychological state^31–33^, also because it is able to give a proxy measurement of respiration, heart-rate, perspiration, and emotional expressions^32,34^. Therefore, the primary aim of the current study is to explore whether facial temperature data can be used to identify blood donors at risk and not at risk of experiencing vasovagal reactions at an early stage of the donation process.

## Results

### Participants

The data was collected from N = 193 blood donors (41% men, control group: n = 70, sensitive group: n = 45, new donors: n = 78). No significant gender (*F*(2) = 2.8, *p* = 0.06) or blood collection center location (*F*(2) = 2.8, *p* = 0.06) differences were found between the groups. There was also no significant difference in age between the groups (*F*(2,192) = 0.870, *p* = 0.417. Control group: *M* = 35.2, *SD* = 13.1, range 20–67; sensitive group: *M* = 33.2, *SD* = 12.5, range 19–65; and new donor group: *M* = 35.2, *SD* = 13.0, range 18–67.

### VVR levels

**Figure 1:**
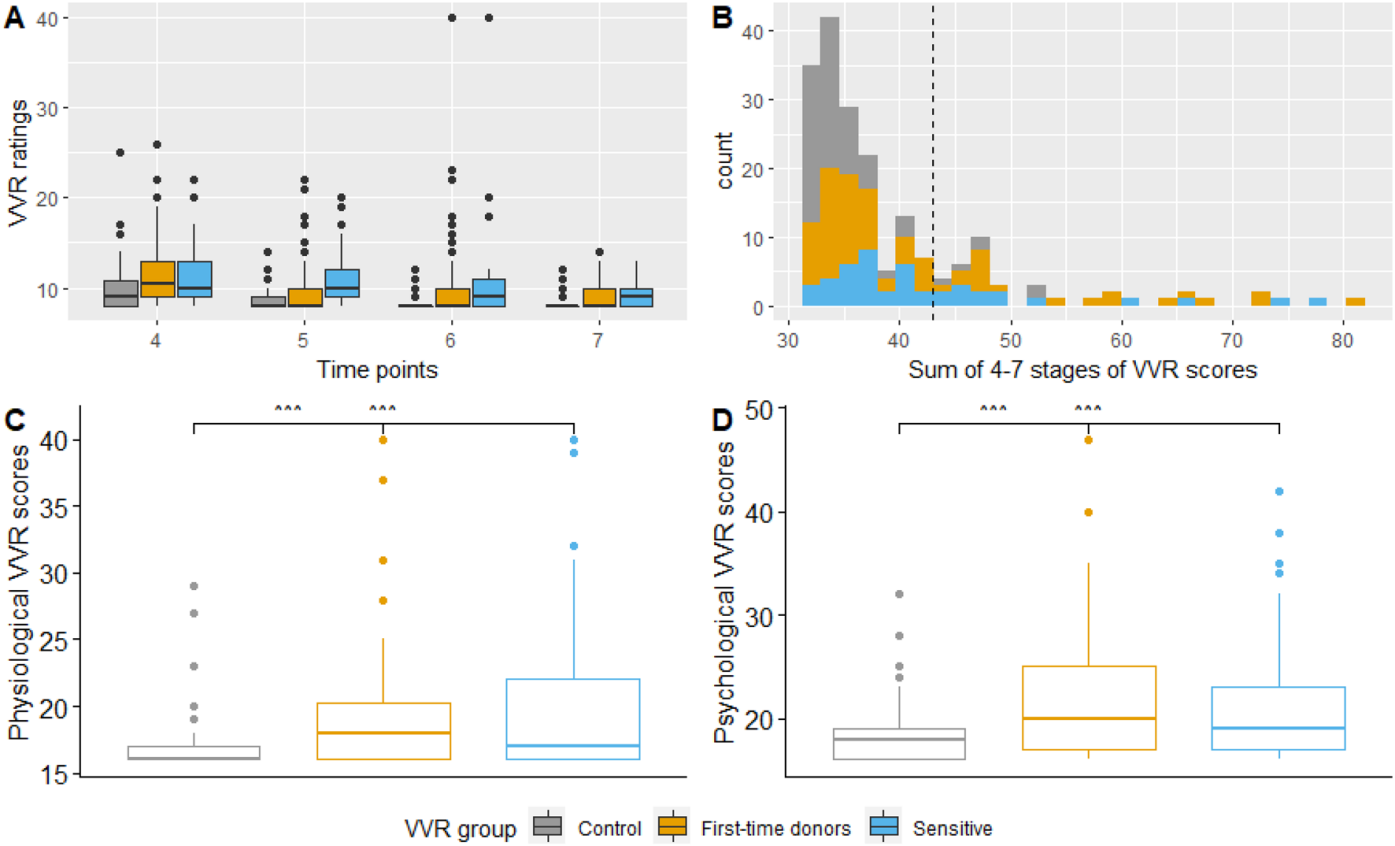
Distribution of self-reported VVR scores during and after the donation procedure. (**A**) Distribution of VVR ratings per time point and group. The dots above the box represent the outliers per group. (**B**) Distribution of total VVR scores. The black dashed line represents the mean of the sample and the cut-off level for the low vs high VVR groups. (**C**,**D**) The spread of psychological, physiological and total VVR scores per group. Physiological VVR symptoms consist of faintness, dizziness, weakness and lightheadedness. Psychological VVR symptoms consist of fear, stress, tension, and nervousness. The line in the box represents the mean of each group and the dots above the box represent the outliers per group.

The VVR scores were positively skewed, reflecting a high proportion of blood donors who reported low VVR scores (*M* = 39.19, *SD* = 9.36, median = 36; min = 32, max = 81, see Fig. 1). Six participants (3%) in the high VVR group experienced symptoms of vasovagal syncope (fainting) which resulted in interference from the donor assistant.

One-way ANOVA's showed a statistically significant main effect of donor group for physiological (*F*(2) = 7.66, *p* = 0.001) and psychological (*F*(2) = 8.5, *p* < 0.001) VVR symptoms. As expected, the control group experienced significantly lower levels of physiological and psychological VVR levels than the experimental (rq, *p* = 0.006 and *p* = 0.001) and new donors (rq, *p* = 0.002 and *p* = 0.002) groups, but no significant differences were found between experimental and new donor groups (respectively, *p* = 0.999 and *p* = 0.81; see Fig. 1).

### Classification groups

For further analysis and classification, the sample was split into a “low VVR score” group (n = 149, VVR score <  = 42, positive class) and a “high VVR score group” (n = 44, VVR level > 42, negative class).

### Cross-correlations between the temperature profiles

A visual representation of the thermal profiles of the facial regions, between the low and high VVR groups during stages 1 and 2, in the waiting area, can be seen in *SI Appendix*, Fig. S1.

For each facial region, we computed the cross-correlation of mean temperature readings over time between the low and high VVR groups. We found the time series of both the low and high groups positively correlated (*SI Appendix*, Fig. S2). When the temperature increased in one group, then it also increased in another group in the areas under the nose, nose, left cheek, and forehead. The most dominant cross-correlations occurred at lag 23 in the area under the nose, at lag 18 in the forehead area, and at lag 16 in the left cheek area indicating that an increase in temperature in these areas in the high VVR group leads to the same increase in low VVR group after less than a second (23, 18 and 16 frames, respectively). Moreover, we found a significant negative cross-correlation in the forehead, right cheek, and nose regions. In these areas, the most dominant cross-correlations occurred at lag -6 in the forehead area, at lag -4 in the right cheek area, and at lag -18 in the nose area, showing that the temperature increases in these areas in the low VVR group are followed by a decrease in the same areas in the high VVR group after less than a second (6, 4 and 18 frames). The strongest correlations were found in the under the nose, right cheek and forehead areas. The cross-correlation between low and high VVR groups of chin area temperature profiles did not reach significance.

### VVR classification results

We applied machine learning algorithms to the extracted time series features in order to classify low and high VVR groups. As the initial model, only the self-reported pre-donation VVR scores were entered. Then we compare the performance of various machine learning algorithms on the extracted facial temperature features, both with and without applying Recursive Feature Elimination on extracted facial temperature features, and with and without self-reported pre-donation VVR scores on the test set (Table 1).

**Table 1:** Machine learning performance values on the testing set for binary classification (positive class = low VVR scores, negative class = high VVR scores) with and without feature selection.

Model		Performance on the test set			
		Precision	Recall	F1	AUC-PR
Decision tree	Pre-donation VVR ratings (N = 2)	0.85	0.69	0.76	0.88
	Facial temperatures + pre-donation VVR ratings (N = 62)	0.92	0.69	0.79	0.92
	Feature-selected facial temperature + pre-donation VVR ratings dataset (RFECV, N = 33)	0.91	0.66	0.76	0.92
	Facial temperature dataset (N = 60)	0.87	0.63	0.73	0.93
	Feature-selected facial temperature dataset (RFECV, N = 22)	0.90	0.56	0.70	0.91
Random forest	Pre-donation VVR ratings (N = 2)	0.85	0.72	0.78	0.90
	Facial temperature dataset with pre-donation VVR ratings (N = 62)	0.87	0.84	0.86	0.94
	Feature-selected facial temperature with pre-donation VVR ratings dataset (RFECV, N = 25)	0.87	0.81	0.84	0.94
	Facial temperature dataset (N = 60)	0.87	0.84	0.86	0.91
	Feature-selected facial temperature dataset (RFECV, N = 48)	0.87	0.81	0.84	0.93
XGboost	Pre-donation VVR ratings (N = 2)	**0.90**	**0.81**	**0.84**	**0.91**
	Facial temperature dataset with pre-donation VVR ratings (N = 62)	0.86	0.78	0.82	0.93
	Feature-selected facial temperature with pre-donation VVR ratings dataset (RFECV, N = 21)	0.90	0.84	0.87	0.94
	Facial temperature dataset (N = 60)	0.90	0.81	0.85	0.94
	Feature-selected facial temperature dataset (RFECV, N = 40)	0.87	0.84	0.86	0.93
Neural network	Pre-donation VVR ratings (N = 2)	0.85	0.69	0.76	0.89
	Facial temperature dataset with pre-donation VVR ratings (N = 62)	0.87	0.84	0.86	0.93
	Feature-selected facial temperature with pre-donation VVR ratings dataset (RFECV, N = 43)	**0.88**	**0.88**	**0.88**	**0.92**
	Facial temperature dataset (N = 60)	0.81	0.69	0.75	0.86
	Feature-selected facial temperature dataset (RFECV, N = 58)	0.79	0.69	0.73	0.85

The best performing models are indicated in bold.

Overall, the best performing model reached the highest F1 score of 0.88 and an AUC-PR score of 0.92, on the dataset combining extracted facial temperature characteristics with pre-donation VVR scores using a neural network classifier. In this model, the temperature characteristics rather than the pre-donation VVR scores were the most important features (see *SI Appendix*, Fig. S4).

**Figure 2:**
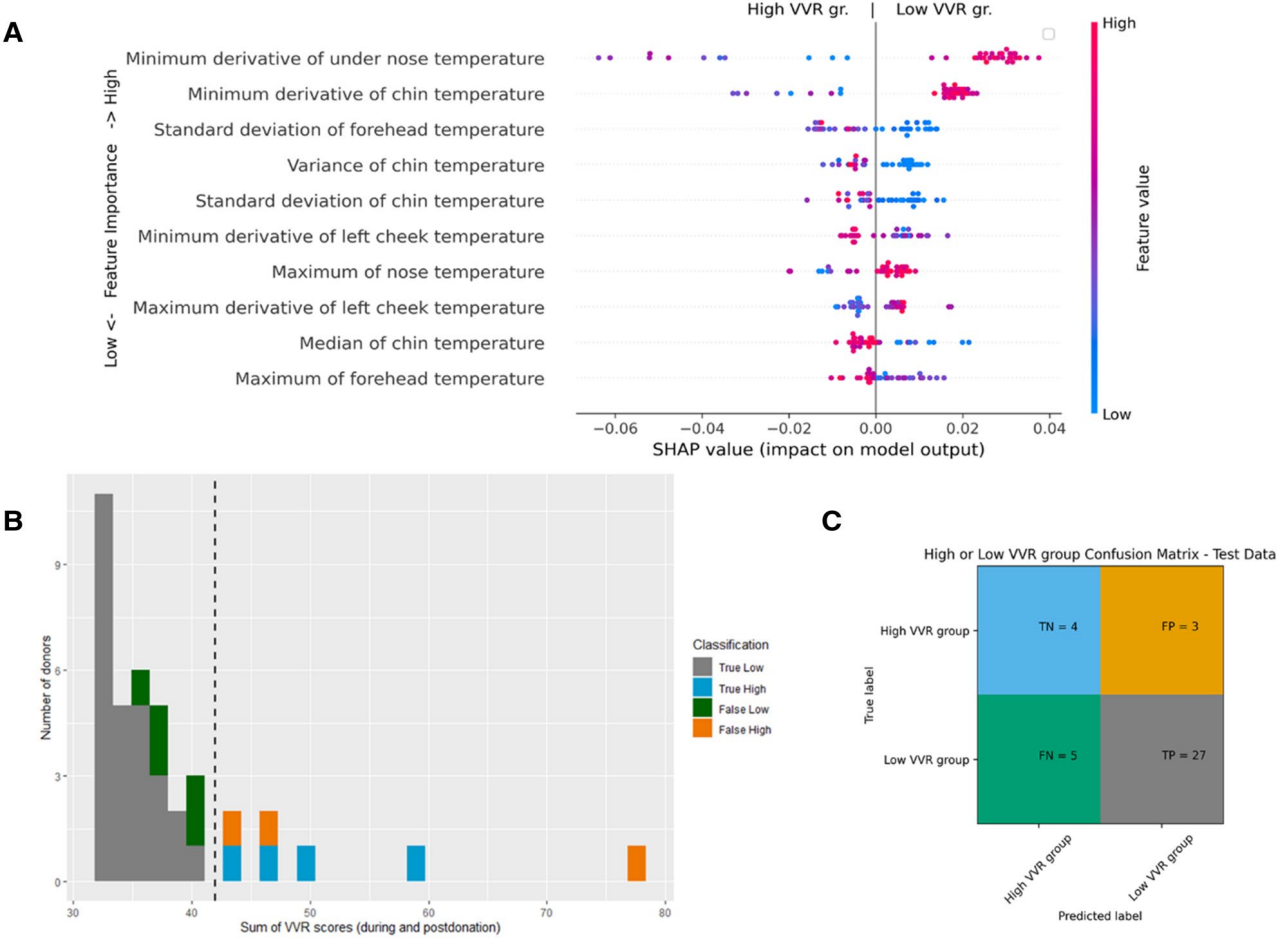
The XGboost performance evaluation on the test set using feature-selected facial temperature dataset (number of features used: 40, number of participants in the test set: 39). The performance evaluation on the test set using XGboost classifier (number of features used: 40, F1 score: 0.86). (**A**) Represents the feature impact on the model output based on the game theoretic SHAP (SHapley Additive exPlanations) approach. The SHAP summary plot combines feature importance (y-axis) with feature effect (x-axis) where each point represents a SHAP value. All features are sorted by importance from the highest to the lowest. A blue color indicates a low and a red color a high value per feature. The negative score on the x-axis is associated with the ‘high-VVR’ group and a positive score on the x-axis is associated with the ‘low-VVR’ group. For example, the higher the minimum derivative value in the ‘under the nose area’ temperature, the higher the chance that the blood donor is classified in the low-VVR group. In contrast, the lower the standard deviation of the temperature in the forehead area, the higher the chance the blood donor will be classified as being in the low-VVR group. (**B**) shows correctly (grey and blue shade) and incorrectly (orange and green shade) classified samples on the test set. (**C**) represents a confusion matrix, which gives a summary of prediction results.

The performance of the models containing only temperature information (so without self-reported VVR scores) were similar and applying feature elimination did not significantly improve the results. For example, precision of decision tree on the test-set was 0.90, however, it has much lower recall of only 0.59 whereas random forest and XGBoost classifier showed a more balanced performance with both precision and recall reaching above 0.80. Since XGboost had slightly higher recall and overall F1 score (Recall = 0.84, F1 score = 0.86, AUC-PR = 0.93) than Random Forest classifier (Recall = 0.81, F1 score = 0.84, AUC-PR = 0.93), we selected this as our best performing classifier. The area under the nose, chin and forehead areas were found to be the most important predictors of this model (see Fig. 2A). The highest minimum derivative of the under the nose and chin area temperatures, as well as a lower standard deviation of the temperature of the forehead, were associated with a higher chance of donors being classified as being in a low VVR group. The best performing model was able to predict participants with a low VVR score with only a few mistakes, however, failed to correctly classify high VVR cases around the mean and some severe cases (see Fig. 2B,C).

## Discussion

The results of this study show that it is possible to classify low and high adverse emotional and physical reactions that occur during a blood donation, based on covert, automatic physiological processes in play in anticipation to the procedure. A neural network and XGboost classifiers trained on anticipatory facial temperature fluctuations, measured with infrared thermal imaging in the waiting room, performed well with an F1 score of 0.88 in classifying low VVR group. Note, the F1 is a measure of how accurately the model performs on new, unseen data and is the harmonic mean of precision and recall, so it balances how many people it captures correctly in the low VVR group. However, the models tend to make more mistakes in high VVR group, which is likely due to the low number of donors who experience a VVR. Just a minority of the donors in the sample experienced very overt, high levels of VVR. Even though this is in line with the previously reported prevalences, with moderate VVRs occurring in between 1.4 and 7% and severe reactions around 0.1% to 0.5% in donors^22,35^, the models would likely improve with a training set including more examples of donors suffering from high levels of VVR.

Donors who experienced VVR symptoms during the donation tend to show higher levels, or greater velocity of, thermal fluctuations around the nose, chin, left cheek and forehead areas. This is in line with previous studies who found decreases in nose tip temperature related to emotional arousal^36,37^ and stress^38^ and perinasal differences related to stress and perspiration^39^ or fear^36^, decrease in forehead temperature associated with fear^40^, stress^38^ and emotional states with low power^41^ and that of cheeks related to stress^38^.

Furthermore, one of the most discriminative areas when it comes to predicting VVR was the area under the nose, which is likely to reflect breathing patterns^34,42^, similar to earlier findings of Trost et al.^43^, which showed an increase in respiratory rate during a virtual blood donation. A more definitive idea of whether these thermal patterns reflect respiration require the comparison to additional respiratory measurements as a gold standard. In the context of aiming to predict the occurrence of VVR it is sufficient to note that this area is indeed a region of interest. Future studies could consider a higher number of facial regions, such as done by^38^.

Previous studies showed that asking the donor to rate their level of fear is a good way to assess the risk of VVR^19,44–46^. Indeed, the best performing models contained both self-reported pre-donation scores and thermal data. However, facial temperature information were more important features than pre-donation scores in those models, with the self-assessed VVR score not surfacing to the top 10 of important features, which is in line with our recent findings in a different study^47^. This is relevant as one of the benefits of using machine learning techniques on video data is that it enables the automatic detection and prediction of vasovagal reactions without having to rely on self-reports or on asking the donor to enter any personal information such as age or gender. This opens up new avenues for prevention, e.g. through biofeedback techniques that are able to support patients in controlling them in a timely manner using for instance easily distributable and user friendly mobile applications such as the AINAR game^48^. The input for such tools could consist of either a ‘click-on’ ITI camera (such as the FLIR ONE) or could rely on photophlesmotography using ‘normal’ video data^40^.

Furthermore, the results corroborate the idea that ITI may be a valid tool for the study of emotions such as arousal, fear and stress^36–41^, especially by looking at features that reflect thermal fluctuations, such as derivatives, mean, median, or standard deviation, rather than considering only increases or decreases in temperature. That said, future studies could assess whether adding other streams of data, including demographic risk factors (e.g. age), other video based features (e.g. facial expressions) or other psychophysiological data (e.g. heart-rate, blood pressure), or contextual factors (e.g. waiting time or even weather conditions^19^ could improve the models to such an extent that their incorporation is advised for clinical application.

## Materials and methods

### Ethics statement

The study was approved by the Ethics Advisory Board of Sanquin and by the REDC of Tilburg University (2019/72). All experiments presented in this paper were performed in accordance with relevant guidelines and regulations. Informed consent was obtained from all participants.

### Participants

Participants were recruited from Sanquin, the not-for-profit organization responsible for the blood and plasma donation chain in the Netherlands. The study took place at three blood collection centers (BCC; Leiden, ‘s-Hertogenbosch, and Zwolle). As the number of previous donations and a prior experience of VVR influence the chance of experiencing VVR at the current donation, three groups of donors were invited to the study to increase the chances of a balanced dataset in terms of prevalence of VVR in the sample. (1) Experienced donors with between 5 and 10 previous donations and no previous experience of vasovagal reactions (control group), (2) experienced donors with 5 to 10 previous donations but who did experience a VVR at their previous donation and who are therefore more likely to experience a VVR again (sensitive group), and (3) first-time donors, who are known to be at higher risk of VVR (new donor group). All blood donors from these locations who received an invitation to donate and who fitted into one of the following three groups were invited to participate.

### Procedure

**Figure 3:**
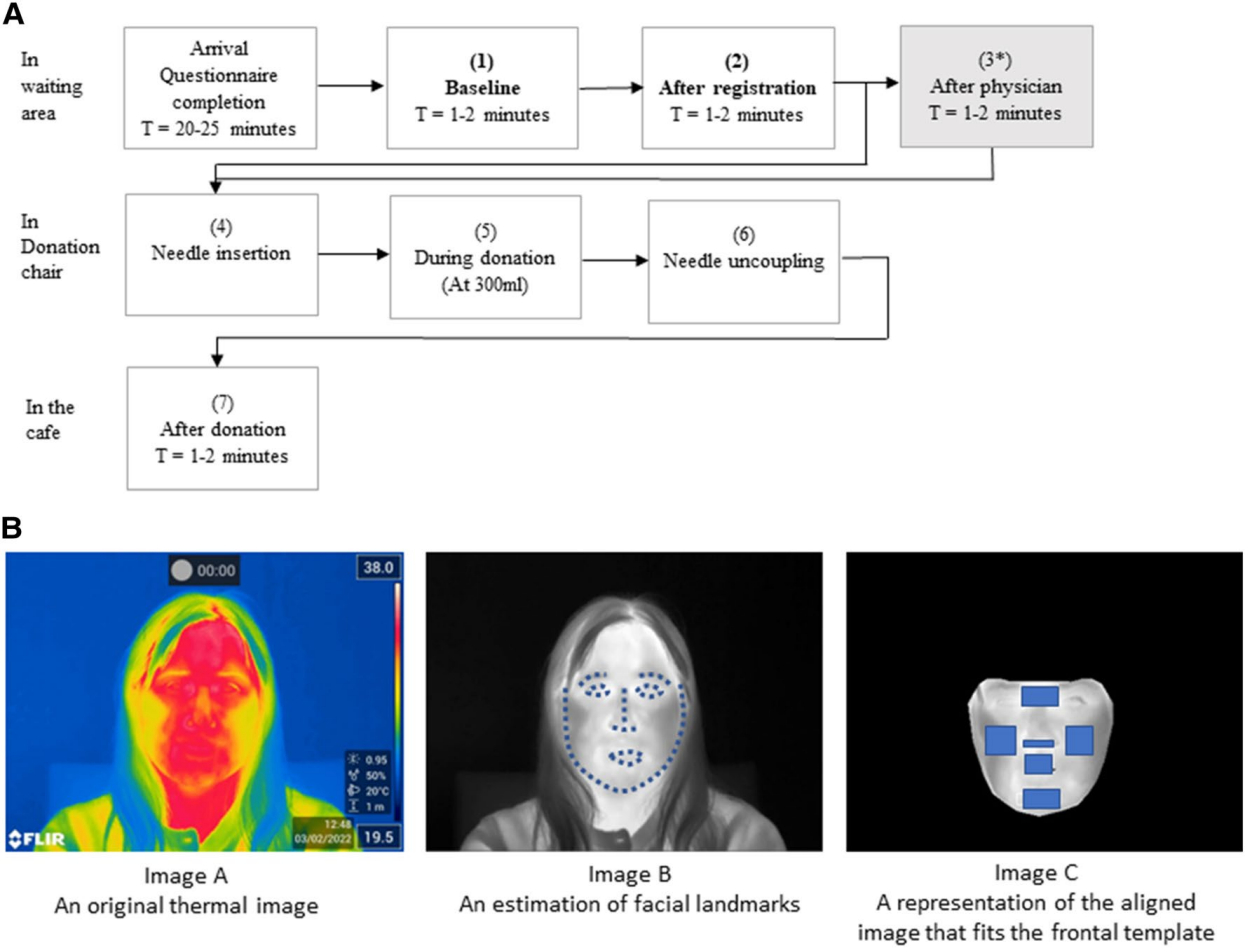
An overview of the blood donation testing procedure and the extraction of mean temperature from the facial regions of interest. (**A**) Overview of the procedure and stages. At each of the seven stages, donors reported their levels of VVR, and an ITI video recording was made for as long as that stage lasted. From stage 4 to stage 6 the recording is continuous and lasts between 5 and 27 min. *The procedure is slightly different per BCC. At two locations, donors are brought directly to the donation chair after the physician check whilst skipping stage 3. Therefore, the ratings of VVR at stage 3 are not included in the total score. The recordings from stages 1 and 2 were combined into one continuous time series. (**B**) Image A represents an original thermal image recorded; Image B represents the same image with facial landmarks fitted; Image C represents the aligned image that fits the frontal template.

Interested donors contacted the data manager for an appointment and received information about the study, including ethical consent information. Donors were requested not to wear glasses during thermographic recordings and to be free from nicotine and caffeine at least 3 h prior to donation. On arrival, participants completed a questionnaire containing items regarding (history and symptoms of) needle fear, and some personality questionnaires. This took between 20 and 25 min to complete. Next, the donors proceeded with the regular blood donation procedure, which consists of several distinct phases: the donor registration, a health check by the donor physician, the actual blood donation, and the cool-down period in the donor cafe. This resulted in seven distinct stages during which ITI and VVR were recorded (see Fig. 3). Thermal recordings from only stage 1 and 2, i.e. before the blood donation, were used for further analysis.

### Materials and measures

#### Thermal imaging

Thermal imaging of the patients was recorded using a FLIR E95 camera, which has a thermal sensitivity of < 40 mK at 30 °C and an infrared resolution of 464 × 348 pixels. The camera was installed on a tripod at a distance of about 1 m from the donor. The camera captured images at 30 frames per second. The room temperature was T = 20.66 °C (SD = 1.4 °C), and the relative humidity was H = 45% (SD = 7.6%). Donors were free to behave as they normally would throughout the procedure.

#### Vasovagal reactions (VVR levels)

At each of the seven stages (see Fig. 3A), participants were asked to rate to what extent they experienced physiological (faintness, dizziness, weakness, lightheadedness) and emotional (fear, stress, tension, and nervousness) reactions, on the Likert scale from 1 (not at all) to 5 (extremely). The ratings of the last four stages^4–7^ were summed, resulting in a score between 32 and 160. This score was subsequently used to distinguish classification target groups of donors experiencing low versus high levels of VVR.

### Thermal data preprocessing

The thermal video data from pre-donation stages 1 and 2 were combined into a continuous time series that served as input for feature selection (N = 2001 frames; see Fig. 3). A visual representation for each frame as well as the raw temperature values per pixel/frame were extracted using the FLIR Research IR tool. To create a time series of temperature, first the facial landmarks were estimated using the Face Alignment Network (FAN)^49^. Then the images were frontalised using a Warp Affine transformation technique^50^ which matches each image to a subject-specific frontal template. Each thermal image was transformed to a frontal one by pasting the calculated triangles from the original image into the template image. The same procedure was completed for both a visual image and a raw temperature file. Each recording lasted for around 2–5 min and frames where the donor was not facing the camera, e.g. positioning themselves or addressing someone else, were removed. As the donors were seated facing the camera in the waiting room during stages 1 and 2, there was minimal deviations in angle or movement.

The following six regions of interest (ROI) were selected: nose, below the nose, cheeks, chin, and the area between the eyes using facial landmark detection. Since each of ROI contains temperature information for each pixel in the ROI, we estimated mean temperature of all pixels in the given ROI, which was further used for ML classification. During pre-donation stages 1–2, the number and angle of the head movements were minimal.

### Time series statistical analysis

To examine the similarities in time series between the ‘low’ and ‘high’ VVR groups, and whether temperature profiles have similar or different patterns, a cross-correlation was used (i.e., a correlation between two time series shifted by k elements relative to one another). Since one of the requirements for applying cross-correlation on time-series data is time series stationarity^51^, the Kwiatkowski-Phillips-Schmidt-Shin (KPSS)^52^ and Augmented Dickey–Fuller (ADF)^53^ tests were applied to check whether the mean and variance of the time series do not vary over time. Detrending or differencing was applied when the observed time series data was non-stationary (see *SI Appendix*, Tables S1, S2).

### Machine learning approach

To find the best performing classifier, four machine learning algorithms were trained to predict whether a donor would have a high or low total VVR score during blood donation: a decision tree, a random forest classifier, an XGBoost and an artificial neural network. As our baseline model, we used the self-reported pre-donation VVR scores from stage 1 and stage 2 as model input (see *SI Appendix*, Fig. S3 for the score distribution).

The features consisted of linear time series characteristics, containing the central properties of the initial dataset using the Tsfresh^54^ python package. In total, 10 features were extracted from each of the facial area temperature profiles such as the sum, variance, standard deviation, maximum-, minimum-, median-, mean-, mean root square, maximum and minimum derivative values/slopes (see *SI Appendix*, Table S3 for feature comparison between low and high VVR groups). This resulted in a total of 60 different features, which were scaled using a standardization technique where the values were centered around the mean with a unit standard deviation.

We tested two sets of features: one using only extracted facial temperature features (N = 60) and one using facial temperature features combined with pre-donation self-reported VVR scores (N = 62). Each time the dataset was split into a training (80%) and test (20%) set. The test set (with only pre-donation VVR ratings) was used to assess the model performance.

We did not have any missing time series, but due to high imbalances in the original dataset (Low VVR group; n = 149 and high VVR group; n = 44), Synthetic Minority Oversampling Technique (SMOTE) was applied to the training set data. SMOTE synthesizes new examples of the minority class based on the nearest neighbor’s technique^55^.

To estimate the generalization performance of the model a nested k-fold cross-validation with an outer k value of 10 and an inner k value of 3 was used. The inner loop was used for feature selection and hyperparameter tuning (we applied GridSearchCV for this purpose, and the hyperparameters explored for each algorithm are presented in *SI Appendix*, Table S4), while the outer loop was used for error estimation. To evaluate the most optimal feature set, we compared the performance of the models on the original dataset and the dataset after the Recursive Feature Elimination with cross-validation (RFECV) was implemented. For RFECV implementation we repeated a nested k-fold cross-validation with GridSearchCV.

Model performance metrics used to evaluate model performance were precision, recall, the F1-score, which is the harmonic mean of precision and recall between 0 and 1, and the AUC-PR, which is the Area Under the Precision-Recall Curve that summarizes a precision-recall curve as the weighted mean of precisions achieved at each threshold value. The higher the score, the better the performance of the model ranging from 0.5 (chance level) to 1.0 being a perfect prediction model.

We used Scikit-Learn^56^, XGboost^57^, Tensorflow^58^ and Keras^59^ in Python to build, tune and evaluate the models. Matplotlib library^60^ in Python and ggplot2^61^ in RStudio were used for visualization. The SHAP (SHapley Additive exPlanations) package in Python^62^, was used to explain the output of the best performing machine learning model.

## Supplementary Information

Supplementary Information.

## Data Availability

The dataset collected during the current study is not publicly available due to participants’ privacy, but preprocessed data are available from the corresponding author on reasonable request.

## References

[1] McLenon J, Rogers MA (2019). The fear of needles: A systematic review and meta-analysis. J. Adv. Nurs..

[2] Taddio A, Ipp M, Thivakaran S, Jamal A, Parikh C, Smart S (2012). Survey of the prevalence of immunization non-compliance due to needle fears in children and adults. Vaccine.

[3] Freeman D, Lambe S, Yu LM, Freeman J, Chadwick A, Vaccari C (2021). Injection fears and COVID-19 vaccine hesitancy. Psychol. Med..

[4] Love AS, Love RJ (2021). Considering needle phobia among adult patients during mass COVID-19 vaccinations. J. Prim. Care Community Health.

[5] Neumann-Böhme S (2020). Once we have it, will we use it? A European survey on willingness to be vaccinated against COVID-19. Eur. J. Health Econ..

[6] Johnson DR, Nichol KL, Lipczynski K (2008). Barriers to adult immunization. Am. J. Med..

[7] McMurtry CM, Riddell RT, Taddio A, Racine N (2015). Far from" just a poke": Common painful needle procedures and the development of needle fear. Clin. J. Pain..

[8] Moya A, Sutton R, Ammirati F, Blanc JJ, Brignole M, Dahm JB (2009). Guidelines: Guidelines for the diagnosis and management of syncope (version 2009): The Task Force for the Diagnosis and Management of Syncope of the European Society of Cardiology (ESC). Eur. Heart J..

[9] Carl Pallais J, Schlozman SC, Puig A, Purcell JJ, Stern TA (2011). Fainting, swooning, and syncope. Prim. Care Companion CNS Disord..

[10] Brignole M, Alboni P, Benditt DG, Bergfeldt L, Blanc JJ, Bloch Thomsen PE (2004). Guidelines on management (diagnosis and treatment) of syncope—update 2004. Europace.

[11] Hoogerwerf MD, Veldhuizen IJT, Tarvainen MP (2018). Physiological stress response patterns during a blood donation. Vox Sang..

[12] Hoogerwerf MD, Veldhuizen IJT, Merz EM (2017). Psychological and hormonal stress response patterns during a blood donation. Vox Sang..

[13] Brinkmann L, Poller H, Herrmann MJ, Miltner W (2017). Initial and sustained brain responses to threat anticipation in blood-injection-injury phobia. NeuroImage Clin..

[14] Ritz T, Meuret AE, Ayala ES (2010). The psychophysiology of blood-injection-injury phobia: Looking beyond the diphasic response paradigm. Int. J. Psychophysiol..

[15] Ayala ES, Meuret AE, Ritz T (2010). Confrontation with blood and disgust stimuli precipitates respiratory dysregulation in blood-injection-injury phobia. Biol. Psychol..

[16] Ehrsson HH, Wiech K, Weiskopf N, Dolan RJ, Passingham RE (2007). Threatening a rubber hand that you feel is yours elicits a cortical anxiety response. Proc. Natl. Acad. Sci. USA..

[17] Lamm C, Nusbaum HC, Meltzoff AN, Decety J (2007). What are you feeling? Using functional Magnetic Resonance Imaging to assess the modulation of sensory and affective responses during empathy for pain. PLoS ONE.

[18] Roelofs K (2017). Freeze for action: Neurobiological mechanisms in animal and human freezing. Philos. Trans. R. Soc. B Biol. Sci..

[19] Suessner S, Niklas N, Bodenhofer U, Meier J (2022). Machine learning-based prediction of fainting during blood donations using donor properties and weather data as features. BMC Med. Inform. Decis. Mak..

[20] Thijsen A, Masser B (2019). Vasovagal reactions in blood donors: Risks, prevention and management. Transfus. Med..

[21] Yoshimoto A, Yasumoto A, Kamiichi Y, Shibayama H, Sato M, Misawa Y, Morita K, Ono Y, Sone S, Satoh T, Yatomi Y (2020). Analysis of vasovagal syncope in the blood collection room in patients undergoing phlebotomy. Sci. Rep..

[22] Almutairi H, Salam M, Alajlan A, Wani F, Al-Shammari B, Al-Surimi K (2017). Incidence, predictors and severity of adverse events among whole blood donors. PLoS ONE.

[23] Wieling W, Thijs RD, Van Dijk N, Wilde AAM, Benditt DG, Van Dijk JG (2009). Symptoms and signs of syncope: A review of the link between physiology and clinical clues. Brain.

[24] Brignole M, Moya A, de Lange FJ, Deharo JC, Elliott PM, Fanciulli A, Fedorowski A, Furlan R, Kenny RA, Martín A, Probst V (2018). Practical instructions for the 2018 ESC guidelines for the diagnosis and management of syncope. Eur. Heart J..

[25] Lorenz TK (2021). Autonomic, endocrine, and psychological stress responses to different forms of blood draw. PLoS ONE.

[26] Torabi P, Rivasi G, Hamrefors V, Ungar A, Sutton R, Brignole M, Fedorowski A (2022). Early and late-onset syncope: Insight into mechanisms. Eur. Heart J..

[27] Donald SJ, McIntyre WF, Dingwall O, Hiebert B, Ponnampalam A, Seifer CM (2019). Is donating blood for the faint of heart? A systematic review of predictors of syncope in whole blood donors. Transfusion.

[28] Merla, A. & Romani, G. L. Thermal signatures of emotional arousal: A functional infrared imaging study. In *Annu Int Conf IEEE Eng Med Biol - Proc.* 247–249 (2007). 10.1109/IEMBS.2007.4352270 18001936

[29] Cross CB, Skipper JA, Petkie D (2013). Thermal imaging to detect physiological indicators of stress in humans. Thermosense Therm. Infrared Appl. XXXV..

[30] Puri, C., Olson, L., Pavlidis, I., Levine, J. & Starren, J. Stresscam: non-contact measurement of users’ emotional states through thermal imaging. In *Conf Hum Factors Comput Syst–Proc*, 1725–1728 https://www.researchgate.net/publication/221516068_StressCam_Non-contact_measurement_of_users’_emotional_states_through_thermal_imaging.

[31] Engert V, Merla A, Grant JA, Cardone D, Tusche A, Singer T (2014). Exploring the use of thermal infrared imaging in human stress research. PLoS ONE.

[32] Cruz-Albarran IA, Benitez-Rangel JP, Osornio-Rios RA, Dominguez-Trejo B, Rodriguez-Medina DA, Morales-Hernandez LA (2019). A new approach to obtain a colour palette in thermographic images. Quant. InfraRed Thermogr. J..

[33] Bhattacharyya A, Chatterjee S, Sen S, Sinitca A, Kaplun D, Sarkar R (2021). A deep learning model for classifying human facial expressions from infrared thermal images. Sci. Rep..

[34] Hu M, Zhai G, Li D (2018). Combination of near-infrared and thermal imaging techniques for the remote and simultaneous measurements of breathing and heart rates under sleep situation. PLoS ONE.

[35] Amrein K, Valentin A, Lanzer G, Drexler C (2012). Adverse events and safety issues in blood donation—A comprehensive review. Blood Rev..

[36] Goulart C, Valadão C, Delisle-Rodriguez D, Caldeira E, Bastos T (2019). Emotion analysis in children through facial emissivity of infrared thermal imaging. PLoS ONE.

[37] Salazar-López E, Domínguez E, Ramos VJ, De la Fuente J, Meins A, Iborra O, Gálvez G, Rodríguez-Artacho MA, Gómez-Milán E (2015). The mental and subjective skin: Emotion, empathy, feelings and thermography. Conscious. Cogn..

[38] Gioia, F. *et al.* Potential physiological stress biomarkers in human sweat. In *2022 IEEE International Symposium on Medical Measurements and Applications (MeMeA)*, 01–06 (IEEE, 2022).

[39] Shastri D, Papadakis M, Tsiamyrtzis P, Bass B, Pavlidis I (2012). Perinasal imaging of physiological stress and its affective potential. IEEE Trans. Affect. Comput..

[40] Ioannou S, Gallese V, Merla A (2014). Thermal infrared imaging in psychophysiology: Potentialities and limits. Psychophysiology.

[41] Zhang M, Ihme K, Drewitz U (2019). Discriminating drivers’ emotions through the dimension of power: Evidence from facial infrared thermography and peripheral physiological measurements. Transport. Res. F: Traffic Psychol. Behav..

[42] Hu MH, Zhai GT, Li D (2017). Synergetic use of thermal and visible imaging techniques for contactless and unobtrusive breathing measurement. J. Biomed. Opt..

[43] Trost Z, Jones A, Guck A, Vervoort T, Kowalsky JM, France CR (2017). Initial validation of a virtual blood draw exposure paradigm for fear of blood and needles. J. Anxiety Disord..

[44] France CR, France JL, Himawan LK, Stephens KY, Frame-Brown TA, Venable GA, Menitove JE (2013). How afraid are you of having blood drawn from your arm? A simple fear question predicts vasovagal reactions without causing them among high school donors. Transfusion.

[45] France CR, France JL, Frame-Brown TA, Venable GA, Menitove JE (2016). Fear of blood draw and total draw time combine to predict vasovagal reactions among whole blood donors. Transfusion.

[46] France CR, France JL, Himawan LK, Lux P, McCullough J (2021). Donation related fears predict vasovagal reactions and donor attrition among high school donors. Transfusion.

[47] Rudokaite J, Ong LLS, Janssen MP, Postma E, Huis in't Veld E (2022). Predicting vasovagal reactions to a virtual blood donation using facial image analysis. Transfusion.

[48] Ainar app (2022) https://apps.apple.com/us/app/ainar-io/id1603046347 (Accessed 14 Apr 2022).

[49] Bulat, A. & Tzimiropoulos, G. How far are we from solving the 2D & 3D face alignment problem? (and a Dataset of 230,000 3D Facial Landmarks). In: *EEE International Conference on Computer Vision (ICCV)*, 1021–1030 http://www.adrianbulat.com/face-alignment/.

[50] Bradski G (2000). The OpenCV library. Dr. Dobb’s J. Softw. Tools.

[51] Kristoufek L (2014). Measuring correlations between non-stationary series with DCCA coefficient. Physica A.

[52] Kwiatkowski D, Phillips PC, Schmidt P, Shin Y (1992). Testing the null hypothesis of stationarity against the alternative of a unit root: How sure are we that economic time series have a unit root?. J. Econ..

[53] Cheung YW, Lai KS (1995). Lag order and critical values of the augmented Dickey-Fuller test. J. Bus. Econ. Stat..

[54] Christ M, Braun N, Neuffer J, Kempa-Liehr AW (2018). Time series FeatuRe extraction on basis of scalable hypothesis tests (tsfresh-A Python package). Neurocomputing.

[55] Chawla NV, Bowyer KW, Hall LO, Kegelmeyer WP (2002). SMOTE: Synthetic minority over-sampling technique. J. Artif. Intell. Res..

[56] Pedregosa F, Varoquaux G, Gramfort A, Michel V, Thirion B, Grisel O (2012). Scikit-learn: Machine learning in Python. J. Mach. Learn. Res..

[57] Chen, T. & Guestrin, C. XGBoost: A scalable tree boosting system. In *Proc ACM SIGKDD Int Conf Knowl Discov Data Min; 13–17-August* 785–794 (2016). https://arxiv.org/abs/1603.02754v3

[58] Abadi, M. *et al.* Tensorflow: Large-scale machine learning on heterogeneous distributed systems. arXiv preprint arXiv:1603.04467. (2016).

[59] Chollet, F. *et al.* Keras. GitHub. Retrieved from https://github.com/fchollet/keras (2015).

[60] Hunter JD (2007). Matplotlib: A 2D graphics environment. Comput. Sci. Eng..

[61] Wickham, H. *ggplot2: Elegant Graphics for Data Analysis* (Springer-Verlag 2016) https://ggplot2.tidyverse.org.

[62] Lundberg, S. M. & Lee, S. I. A unified approach to interpreting model predictions. In *Neural Information Processing Systems (NIPS 2017)* (2017).

