# Supplementary material for: Predicting vasovagal reactions to needles with anticipatory facial temperature profiles
Source: Sci Rep. 2023 Jun 14;13:9667. doi: 10.1038/s41598-023-36207-z (PMC10266310; doi:10.1038/s41598-023-36207-z)
Supplement: Supplementary file 1 — Supplementary Information. [file 41598_2023_36207_MOESM1_ESM.pdf]

SUPPLEMENTARY MATERIALS

Table S1. Evaluation of time series stationarity for each facial region for both the high and low VVR groups.

Facial area	VVR Score group	Kwiatkowski-Phillips-Schmidt-Shin (KPSS) test for level or trend stationarity.			Augmented Dickey–Fuller (ADF) t-statistic test for unit root.		
		KPSS Level	Truncation lag parameter	P value	Augmented Dickey-Fuller	Lag order	P value
Under nose	Low	14.35	8	0.01	-4.95	12	0.01
	High	10.65	8	0.01	-3.41	12	0.05
Forehead	Low	11.12	8	0.01	-6.46	12	0.01
	High	1.06	8	0.01	-4.92	12	0.01
Left cheek	Low	9.66	8	0.01	-5.53	12	0.01
	High	1.77	8	0.01	-3.59	12	0.03
Right cheek	Low	18.52	8	0.01	-7.27	12	0.01
	High	13.74	8	0.01	-7.13	12	0.01
Chin	Low	11.46	8	0.01	-6.14	12	0.01
	High	0.98	8	0.01	-5.71	12	0.01
Nose	Low	18.45	8	0.01	-5.58	12	0.01
	High	15.90	8	0.01	-4.84	12	0.01

Table S2. Evaluation of time series of each facial region from two groups (1 = low VVR score group, 2 = high VVR score group) stationarity after detrending or differencing was applied.

Facial area	VVR Score group	Kwiatkowski-Phillips-Schmidt-Shin (KPSS) test for level or trend stationarity.			Augmented Dickey–Fuller (ADF) t-statistic test for unit root.		
		KPSS Level	Truncation lag parameter	P value	Augmented Dickey-Fuller	Lag order	P value
Under nose	Low	0.19	8	0.1	-9.02	10	0.01
	High	0.39	8	0.08	-7.4	9	0.01
Forehead	Low	0.44	8	0.06	-9.1	9	0.01
	High	0.29	8	0.1	-8.1	10	0.01
Left cheek	Low	0.27	8	0.1	-8.77	9	0.01
	High	0.02	8	0.1	-7.896	9	0.01
Right cheek	Low	0.15	8	0.1	-8.56	10	0.01
	High	4.3	8	0.1	-9.46	9	0.01
Chin	Low	0.21	8	0.1	-7.9	9	0.01
	High	0.46	8	0.05	-9.97	10	0.01
Nose	Low	0.086	7	0.1	-6.73	7	0.01
	High	0.06	7	0.1	-8.07	7	0.01

Table S3. Non-parametric Mann–Whitney U test was conducted to compare differences for extracted facial temperature features (N = 60) between low and high VVR groups.

Facial area	Temperature feature	Mann-Whitney U	P value
Left cheek	Sum	3209	0.83
	Median	3294	0.96
	Mean	3212	0.84
	Standard Deviation	3728	0.17
	Variance	3728	0.17
	Root mean square	3222	0.86
	Maximum	3384	0.75
	**Minimum**	**2324**	**0.002**
	**Max derivative**	**3940**	**0.04**
	**Min derivative**	**2605**	**0.04**
Right cheek	**Sum**	**2296**	**0.026**
	**Median**	**2317**	**0.003**
	**Mean**	**2297**	**0.0026**
	Standard Deviation	3583	0.35
	Variance	3583	0.35
	**Root mean square**	**2303**	**0.0028**
	**Maximum**	**2571**	**0.03**
	**Minimum**	**2332**	**0.0014**
	**Max derivative**	**4222**	**0.0038**
	**Min derivative**	**2365**	**0.005**
Nose	Sum	2971	0.35
	Median	3030	0.45
	Mean	2971	0.35
	**Standard Deviation**	**4008**	**0.025**
	**Variance**	**4008**	**0.025**
	Root mean square	2978	0.36
	Maximum	3227	0.88
	**Minimum**	**2389**	**0.002**
	**Max derivative**	**4165**	**0.006**
	**Min derivative**	**2438**	**0.01**
Chin	Sum	3287	0.98
	Median	3301	0.95
	Mean	3290	0.97
	**Standard Deviation**	**3948**	**0.04**
	**Variance**	**3948**	**0.04**
	Root mean square	3296	0.96
	Maximum	3335	0.86
	**Minimum**	**2297**	**0.001**
	**Max derivative**	**4210**	**0.004**
	**Min derivative**	**2410**	**0.0077**
Under nose	Sum	3182	0.77
	Median	3226	0.87
	Mean	3183	0.77
	Standard Deviation	3725	0.17
	Variance	3725	0.17
	Root mean square	3184	0.77
	Maximum	3147	0.69
	**Minimum**	**2285**	**0.0008**

	**Max derivative**	**4221**	**0.0038**
	**Min derivative**	**2276**	**0.002**
Forehead	Sum	3243	0.92
	Median	3251	0.94
	Mean	3244	0.92
	**Standard Deviation**	**4076**	**0.014**
	**Variance**	**4076**	**0.014**
	Root mean square	3244	0.92
	Cheek maximum	3524	0.45
	**Minimum**	**2255**	**0.0006**
	**Max derivative**	**4284**	**0.002**
	**Min derivative**	**2355**	**0.0046**

Table S4. Hyperparameter tuning using GridSearchCV for each machine learning algorithm.

**ML algorithm**	**Parameter**	**Values/Range**
XGBoost	Learning rate	[0.0001, 0.001]
	Max depth	[1, 2, 3, 4]
	Subsample	[0.6, 0.8]
	Col sample by tree	[0.5, 0.75, 1]
Decision Tree	Max depth	[2, 3, 5, 10, 20]
	Min samples leaf	[5, 10, 20, 50, 100]
	Criterion	[‘gini’, ‘entropy’]
Random Forest	Max depth	[2, 3, 5, 10, 20]
	Min samples split	[0.1, 0.3, 1]
	Min samples leaf	[1, 3, 10]
	Criterion	[‘gini’, ‘entropy’]
Neural networks	Batch size	[32, 64]
	Number of epochs	[50, 100, 200]

Figure S1: A visual representation of the thermal profiles of the facial regions between the low and high VVR groups during stage 1 and 2.

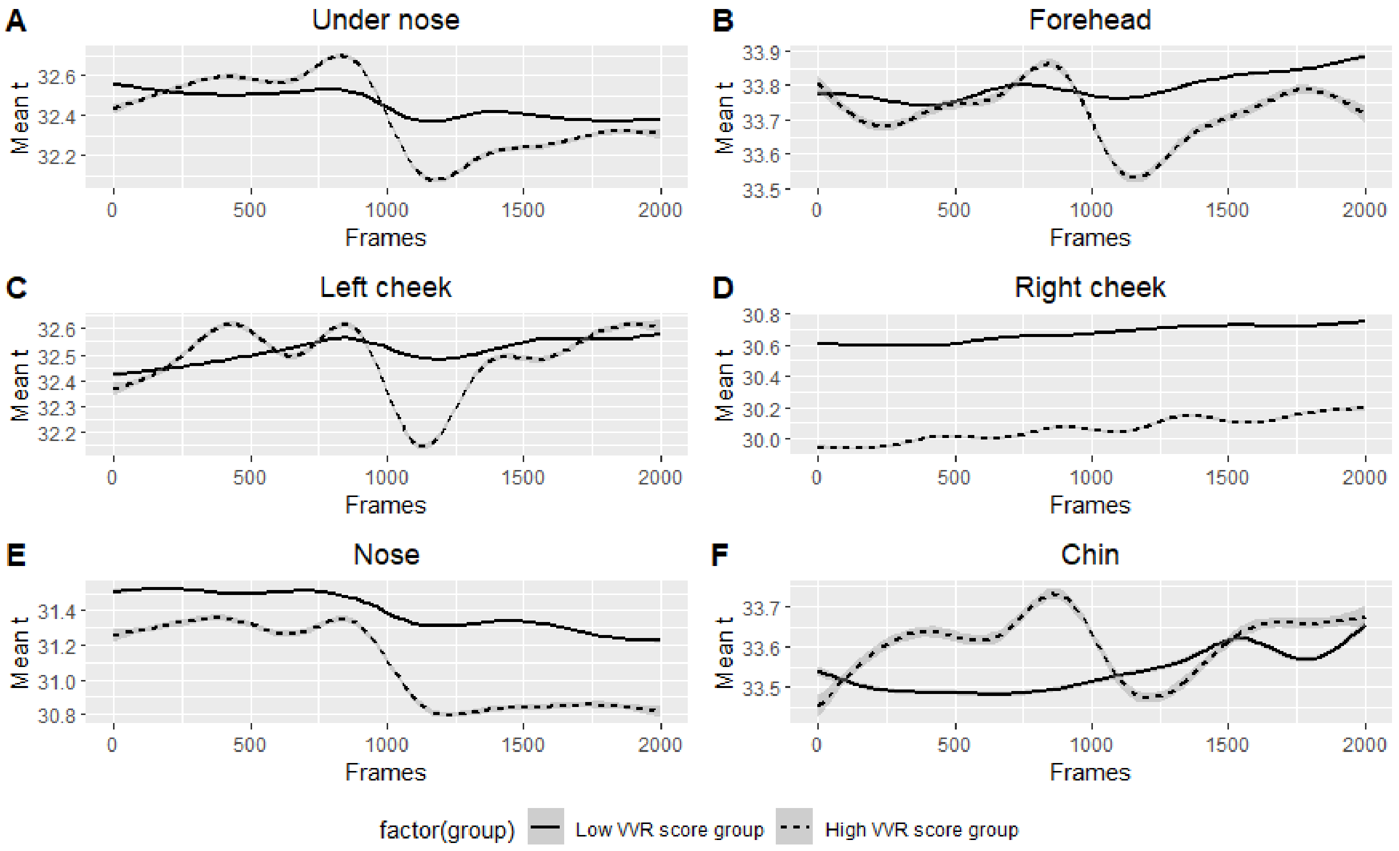

Note: Smoothed mean temperature observed at six facial regions in the waiting area (N = 2001 frames) from the low VVR and high VVR score group.

Figure S2. Cross-correlation between low and high VVR groups at six facial regions.

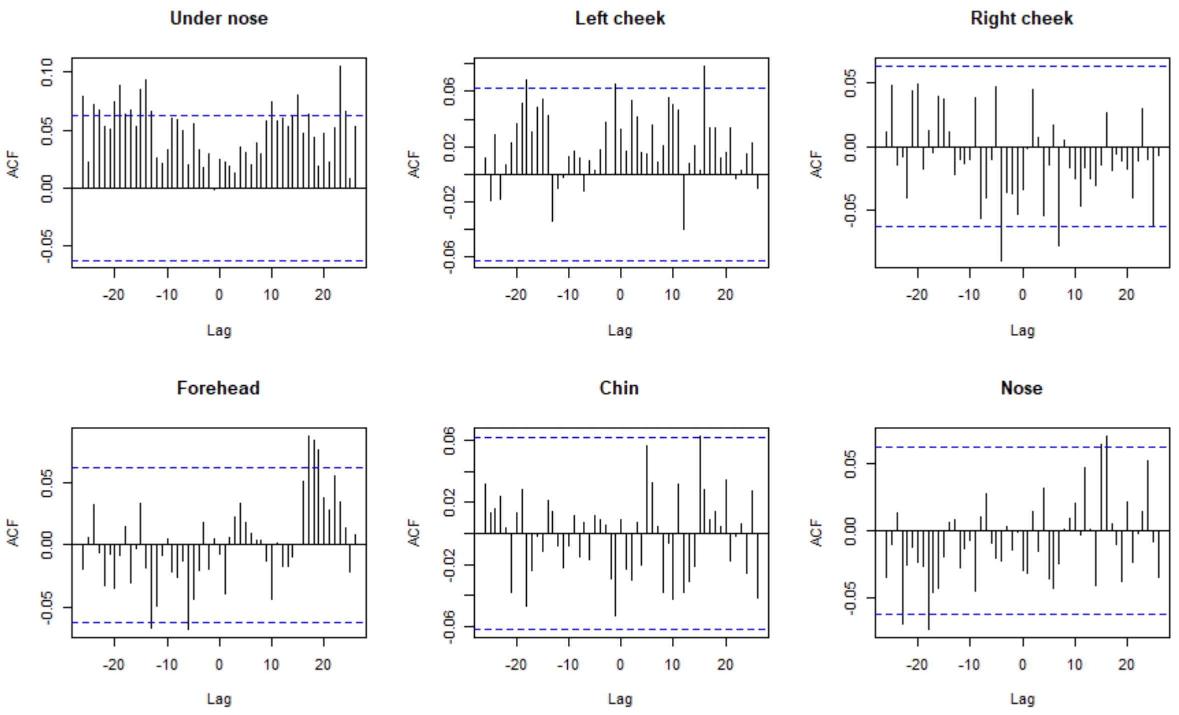

Note: The dashed blue line indicates the significant threshold. Since all of the time series was non-stationary (See Table S2), detrending or differencing was applied on the data and stationarity was checked again using both tests (See Table S3).

Figure S3. Distribution of VVR ratings per time point and group. The dots above the box represent the outliers per group.

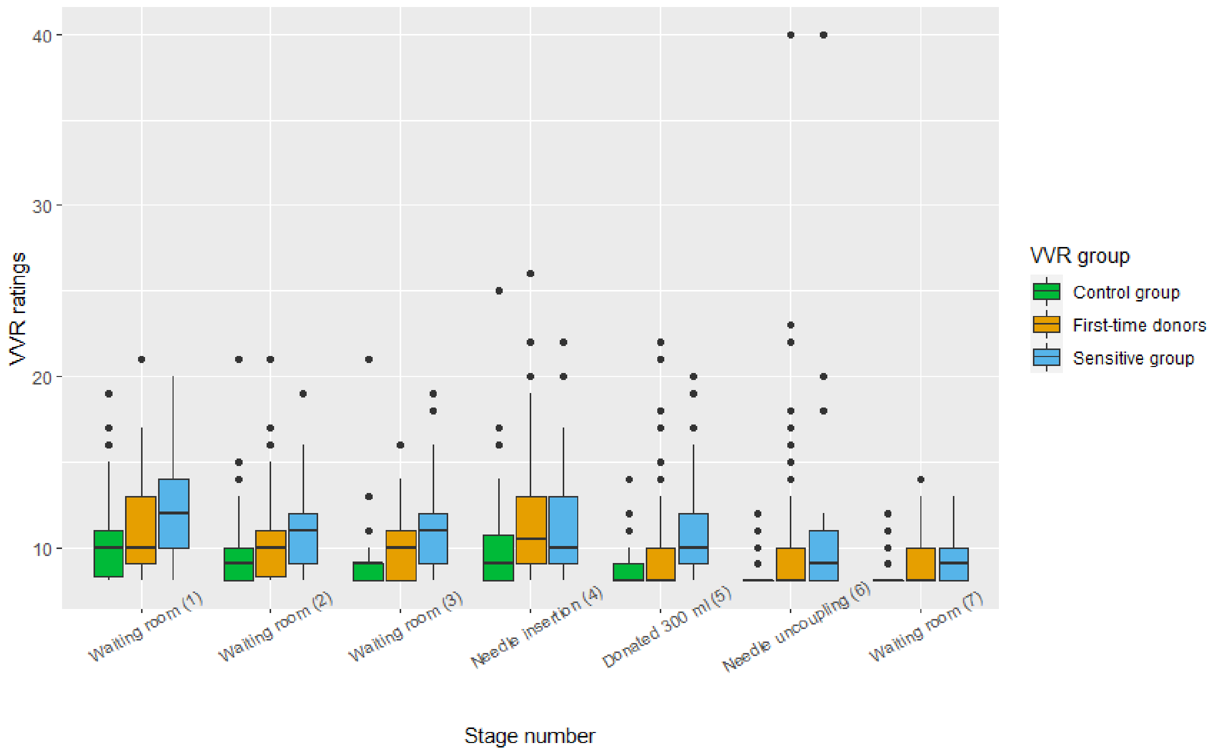

Figure S4. The neural network performance evaluation on the test set using feature-selected facial temperature dataset and pre-donation VVR scores (Number of features= 43).

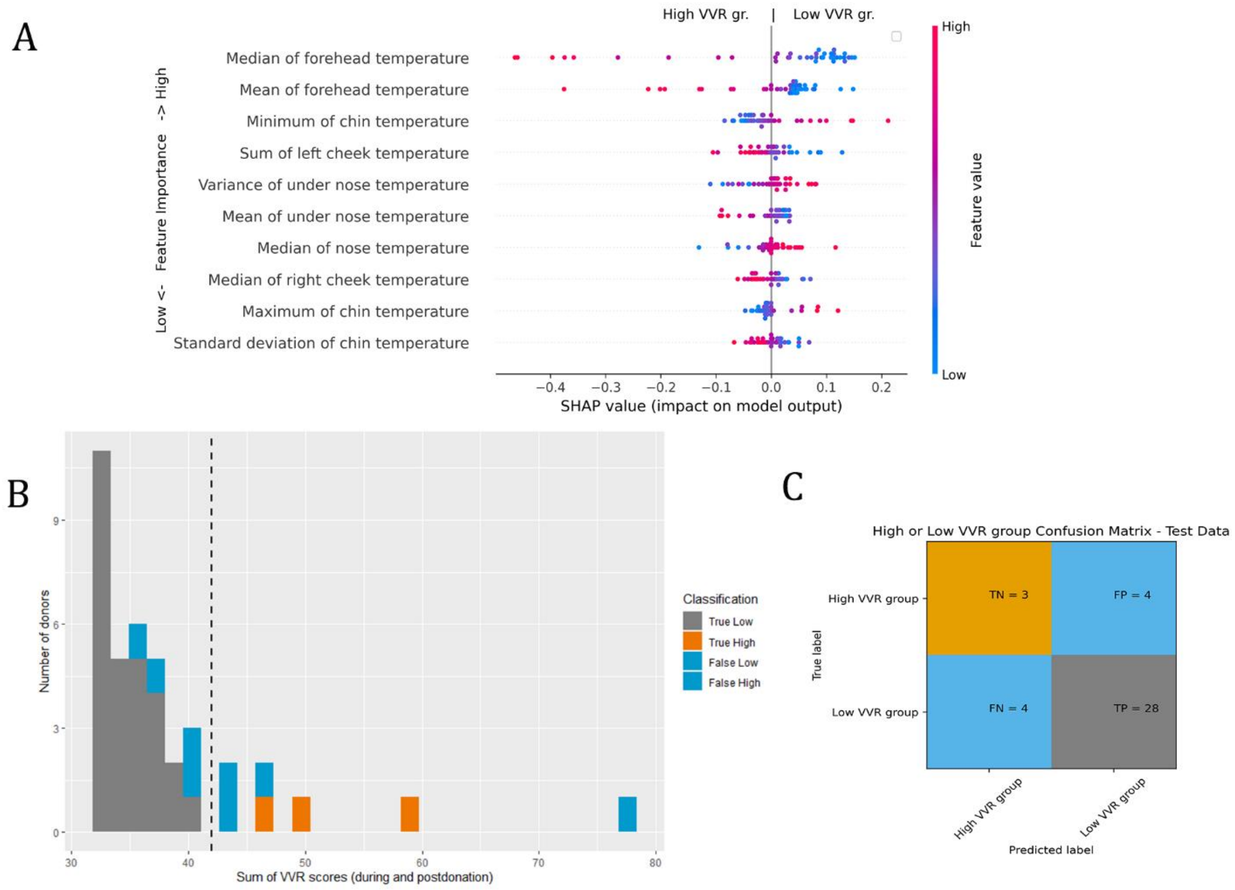

Note: The performance evaluation on the test set using neural network classifier (N = 39, F1 = 0.88). <u>Figure A</u> represents feature impact on the model output based on the game theoretic approach SHAP (SHapley Additive exPlanations). The SHAP summary plot combines feature importance (y-axis) with feature effect (x-axis) where each point represents a SHAP value. All features sorted by importance from the highest to the lowest. Blue color indicates low values and red color indicates high values of the given temperature features. The negative score on the x-axis is associated with 'high-VVR' group and positive score on the x-axis is associated with 'low-VVR' group. For example, the <u>higher</u> median temperature of the forehead, the higher chance of blood donor being classified being in a high-VVR group. In contrast, the <u>lower</u> median temperature of the forehead area, the higher chance the blood donor to be classified as being in a low-VVR group. <u>Figure B</u> shows correctly (grey and orange

shade) and incorrectly (blue shade) classified samples on the test set, and Figure C represents a confusion matrix, which gives a summary of prediction results.
